# Long-Term Outcomes of Personalized Stereotactic Ablative Brachytherapy for Recurrent Head and Neck Adenoid Cystic Carcinoma after Surgery or External Beam Radiotherapy: A 9-Year Study

**DOI:** 10.3390/jpm11090839

**Published:** 2021-08-26

**Authors:** Yi Chen, Jinzhao Dai, Yuliang Jiang, Zhe Ji, Ping Jiang, Haitao Sun, Fei Xu, Junjie Wang

**Affiliations:** 1 Department of Radiation Oncology, Peking University Third Hospital, Beijing 100191, China; yichen@bjmu.edu.cn (Y.C.); yuliangjiang@bjmu.edu.cn (Y.J.); aschoff@bjmu.edu.cn (Z.J.); jiangping@bjmu.edu.cn (P.J.); sunht_puth@163.com (H.S.); xuf_puth@163.com (F.X.); 2 Department of Nuclear Medicine, Qingdao Central Hospital, Qingdao 266300, China; daijz_sdqd@163.com

**Keywords:** adenoid cystic carcinoma, head and neck neoplasm, Iodine-125, stereotactic ablative brachytherapy, interstitial brachytherapy

## Abstract

The management of recurrent head and neck adenoid cystic carcinoma (HNACC) remains a problematic challenge. This study aims to evaluate the long-term outcomes of personalized stereotactic ablative brachytherapy (SABT) as a salvage treatment for recurrent HNACC after surgery or external beam radiotherapy (EBRT). 21 patients with recurrent HNACC after surgery or EBRT successfully underwent iodine-125 (I-125) seed SABT from May 2011 to November 2019. The objective response rate (ORR), disease control rate (DCR), local control time (LCT), overall survival (OS), symptomatic relief and adverse events (AEs) were analyzed. Following SABT, the ORR and DCR were 85.7% and 100%, respectively. The 3-, and 5-year LCT rates were 68.8% and 55.1%, respectively, and the 3- and 5-year OS rates were 85.9% and 66.2%, respectively. Furthermore, univariate analyses showed that higher D90 (>137.1 Gy) was a strong positive prognostic factor of LCT (*p* < 0.05). The pain disappeared in one patient 3 months after SABT and partial pain improvement was observed in nine patients 1 to 6 months after SABT. Additionally, dyspnea was relieved in one patient with the tumor involving the trachea. The major AEs were mild intraoperative hemorrhage and skin/mucosal toxicities which were generally graded ≤2 and well-tolerated. Personalized SABT was an effective and safe alternative option for recurrent HNACC after the previous failure of surgery or EBRT. The parameter of D90 may influence the local control.

## 1. Introduction

Head and neck adenoid cystic carcinoma (HNACC) is a rare cancer, accounting for approximately 1% of all head and neck malignancies. It usually arises from the major and the minor salivary glands [1], also arising from other sites including the nasal and paranasal sinuses, trachea and larynx, lacrimal and ceruminous glands [2].

The primary therapy for HNACC remains wide surgical resection followed by adjuvant postoperative radiotherapy that significantly improves the survival [3,4,5]. Unfortunately, clinicians assume that “cure is probably impossible for HNACC” due to its high risk of local recurrence and distant metastasis. Even though occasional favorable survival outcomes are reported (5-year overall survival (OS) rate of 92% in the Australia study [6], 10-year OS rates of 63.7% in the Japan study [7] and 20-year OS rate of 40% in the England study [8]), the survival rates continue to decline, even until 30 years.

Repeated local recurrence, actually, is the leading cause of treatment failure in HNACC. The local recurrence rate was 54% in the Australia study [6], and it reached 100% at 30 years in the Jones’ study [8]. To handle the local recurrence of HNACC after initial surgery and/or external beam radiotherapy (EBRT) is a problematic challenge. Re-resection is rarely an option for most patients in clinical practice due to the difficulty of complete resection, high risk of complications, functional preservation, cosmesis and patients’ refusal after suffering several prior operations. Worse still, it is rather challenging to deliver high enough doses to the recurrent disease in the previously irradiated area using re-EBRT due to the dose limitation of the adjacent normal tissues. The role of chemotherapy for recurrent HNACC remains controversial. Therefore, new therapeutic strategies are urgently needed.

In response, personalized stereotactic ablative brachytherapy (SABT) using low dose rate (LDR) or high dose rate (HDR) interstitial brachytherapy may resolve this puzzle. An increasing number of studies demonstrate that SABT, characterized by delivering high doses to the tumor and well sparing the adjacent normal tissues, is an effective alternative for most recurrent cancers [9,10,11,12,13,14,15,16,17,18,19,20,21,22,23]. Previous studies in our center have shown that SABT is an effective and safe option for recurrent head and neck squamous carcinoma and soft tissue sarcoma [9,10], recurrent cervical cancer [13], recurrent retroperitoneal lymphatic metastasis [24] and rectal cancer [16]. SABT of iodine-125 (I-125) seed implantation brachytherapy is recommended by NCCN Guidelines of Rectal Cancer 2015.v2 as a salvage treatment for locally recurrent rectal carcinoma [25]. Moreover, the SABT of HDR interventional brachytherapy is also recommended by ESMO clinical guidelines as a ablative treatment for hepatocellular carcinoma [26]. This study intends to report the long-term outcomes of recurrent HNACC patients treated with I-125 seed SABT to provide more advice for the treatment of recurrent HNACC.

## 2. Results

### 2.1. Patient Characteristics

A total of 23 patients were considered for SABT over 9 years, while two patients were finally excluded: one with a high tendency of bleeding and the other with a tumor involving the facial skin and leading ulceration. Finally, the SABT was performed with the CT guided freehand implantation technique for eight patients. Furthermore, a CT guided and 3D printed non-coplanar template (3D-PNCT) assisted technique was employed for other 13 patients. The characteristics and SABT information of the 21 patients are shown in Table 1.

### 2.2. Symptom Relief

The pain disappeared in one patient 3 months after seeding and partial pain improvement was observed in nine patients 1 to 6 months after seeding. Additionally, dyspnea was relieved in one patient with the tumor invading the trachea.

### 2.3. Treatment Response

**Figure 1:**
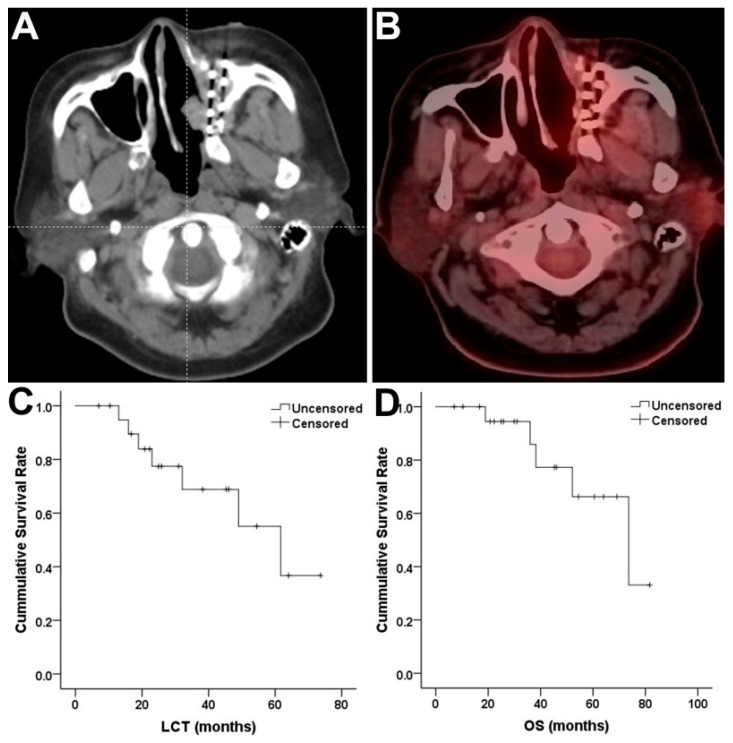
Short-term and long-term efficacy of stereotactic ablative brachytherapy (SABT) for recurrent head and neck adenoid cystic carcinoma (HNACC). (**A**,**B**) showed the complete response of one patient 3 months after SABT. (**C**,**D**) showed the LCT and OS of all patients.

Four patients achieved CR (Figure 1), 14 patients achieved PR, three patients achieved SD and no patients developed progression within 6 months after SABT with the ORR of 85.7% and the DCR of 100%.

### 2.4. LCT

The median follow-up period was 37.5 months (range, 12–84 months). During the follow-up, 7 patients developed local recurrence. The median LCT was 61.7 months with three- and five-year LCT rates of 68.8% and 55.1% respectively (Figure 1).

### 2.5. OS

Of 21 patients, five died: one suffocated by severe hemorrhage, one died from metastasis and the other died from local progression. There was no treatment-related death. The median OS was 73.6 months with 3- and 5-year OS rates of 85.9% and 66.2%, respectively (Figure 1).

### 2.6. Univariate Analyses of LCT and OS

LCT and OS were important parameters reflecting the efficacy; therefore, factors possibly influencing these two outcomes were analyzed.

**Figure 2:**
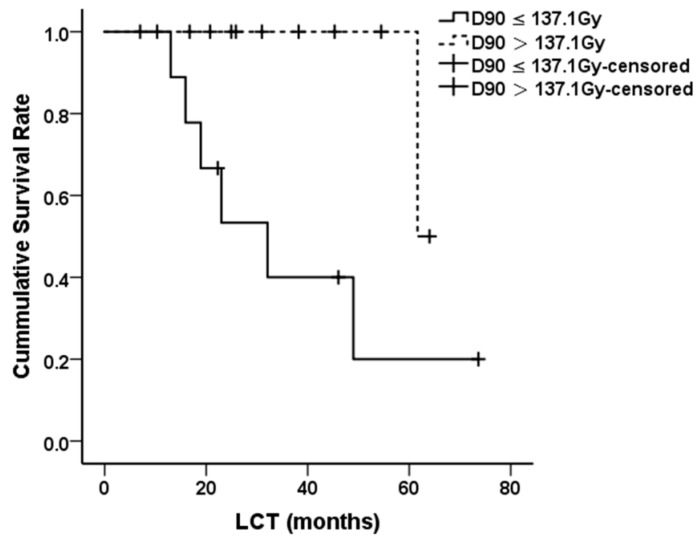
Local control time (LCT) of patients with different D90.

Univariate analyses for LCT (Table 2) showed that the patients with higher D90 (>137.1 Gy) had a more favorable LCT than those with lower doses (61.7 months versus 32.1 months, *p* = 0.048) (Figure 2). Multivariate analyses were not carried out due to the low event rate.

Univariate analyses indicated no independent factors for OS in this case series (Table 3).

### 2.7. AEs

The puncture-related and radiation-related AEs were reviewed. Mild intraoperative hemorrhage was observed in all cases while no moderate and severe hemorrhage occurred during and after operation. No patient developed postoperative infection. Six patients developed Grade 1 skin reaction and one developed Grade 2 skin reaction. Grade 1 mucosal reaction was observed in two patients.

## 3. Discussion

In this study, most of the patients (12/21) underwent surgical resection and neo-adjuvant or adjuvant EBRT as an initiative therapy before recurrence. Some patients even received multiple times of resection and EBRT. It is a challenge to deal with the local recurrent disease after the failures of various therapies. Patients in the present study were deemed as unfit for surgery in the light of the surgeon’s consultation or personal refusal. Additionally, re-EBRT, due to the dose limitation of the normal structures or individual choice, was not recommended.

SABT, theoretically, can deliver a high enough dose to the tumor and spare the adjacent normal tissues considering its better conformity, more rapid dose fall-off and lower dose rate compared to EBRT [27]. The facts bear out the theory. SABT has been recommended as one of the standard care methods for prostate carcinoma by the guidelines for years. Furthermore, it is also recommended by ESMO clinical guidelines as an ablative treatment for hepatocellular carcinoma [26]. Previous studies, in increasing numbers, further demonstrate its efficacy and safety in various cancers, especially for head and neck cancer after multiple therapies [9,10,11,12,13,14,15,16,17,18,19,20,21,22,23]. The SABT is constantly evolving, especially the invention of 3D-PNCT that significantly improves the accuracy, efficacy, security and productivity of the SABT. Therefore, SABT may be a preferred alternative for recurrent HNACC.

In this study, ORR was 85.7% and DCR was 100% in the patients treated with I-125 seed SABT. Moreover, the median LCT reached 61.7 months with the 3- and 5-year LCT rates of 68.8% and 55.1%, respectively, which indicated a good short-term efficacy of SABT guided by 3D-PNCT and/or CT. These favorable results are consistent with previous studies. Ashamalla et al., in 2002, even tried to use ^198^Au seeds to treat head and neck cancer and reported an average progression-free survival of 52 months in recurrent HNACC (4 patients) although the seeds were implanted with freehand without CT guidance and template assistance [28]. Zhang et al. reported a 5-year LCT rate of 57.3% for recurrent HNACC (29 patients) [29]. In Zhang’s study, seed implantation was assisted by a 3D-printing coplanar template (3D-PCT) and guided by CT. Different from the above studies, in our study most cases of SABT were assisted by the 3D-PNCT and guided by the CT. The results from previous studies and ours suggested that SABT had a good efficacy for HNACC. Interestingly, the local control seemed better in our and Zhang’s study than in Ashamalla’s study, which was probably attributed to the application of template and CT. Our previous work indicated that template assistance significantly improved the accuracy and efficacy of SABT [30]. Additionally, 3D-PNCT is superior to the 3D-PCT in some aspects. Ji et al. reported that the dose in target volume margin was higher, needles were fewer, and safety was higher in the plan assisted by 3D-PNCT than in the plan assisted by 3D-PCT for lung cancer [31]. Qu et al. pointed out that both 3D-PNCT and 3D-PCT plans for patients with pelvic recurrence of gynecological malignancies had similar dosimetry, but 3D-PNCT improved the security of SABT [32]. This study, as far as we know, is probably the first to report the long-term outcomes of the I-125 seed SABT assisted by 3D-PNCT and CT for recurrent HNACC after the failure of previous surgery and/or EBRT. As to long-term survival, our study showed that the median OS was 73.6 months with the 3- and 5-year OS rates of 85.9% and 66.2%, respectively. Zhang et al. reported similar favorable results [29].

As the therapeutic experience of the recurrent HNACC is scarce, prognostic factors remain unclear. Univariate analyses showed that the LCT was significantly prolonged in patients with a higher target dose (D90 > 137.1 Gy), which suggested that the local control may be improved if the prescribed dose is raised. Ji et al. [9] and Qu et al. [13] have reported similar results in recurrent head and neck cancer and cervical cancer. Our previous work reconfirmed this in retroperitoneal lymph node metastasis [24]. Therefore, exploring an appropriate prescribed dose of SABT for recurrent HNACC is of crucial clinical significance.

Most of the patients in this study obtained pain relief after implantation. In addition, one patient was relieved of severe dyspnea. Similar results are observed in previous studies [29]. These consistent results suggest that SABT is effective in symptom relief.

The only recorded AEs were mild intraoperative hemorrhage and skin/mucosal toxicities generally graded ≤ 2 and well-tolerated. Previous studies also observed low toxicities of SABT as a salvage treatment for recurrent head and neck cancer. Ji et al. reported that grade 3 skin/mucosal toxicities were 7.9% and grade 4 were 2%. Jiang et al. [33] explored the side effects of SABT in the treatment of recurrent head and neck cancer showing that 14.4% of patients had grade 1/2 skin/mucosal toxicities, while no patients suffered from grade 3/4 skin/mucosal reaction. Bussu et al. reported that transient paralysis of the sixth cranial nerve was the only recorded AE in head and neck cancer treated with SABT of HDR interstitial brachytherapy [19]. Strnad et al. reported that the soft tissue or bone necrosis rates were 17.3% and 9.6%, respectively, in head and neck malignancies (only 3% of the cases needed surgical treatment) after SABT with/without other therapeutic modalities [21]. Teudt and Kovacs et al. reported that the safety of perioperative SABT in recurrent or advanced head and neck metastases was good (the acute and late toxicities were both graded ≤ 2) [23]. Conversely, the toxicities of re-EBRT seemed more severe in head and neck malignancies compared to SABT. De Crevoisier et al. [34] reported that the Grade 3 and Grade 4 mucositis were 32% and 14%, respectively, for re-EBRT in head and neck carcinoma. Furthermore, RTOG 9610 reported that the patients treated with re-EBRT combined with chemotherapy developed grade 3 skin/mucosal toxicities in 34.2% of the patients and grade 4 skin/mucosal toxicities in 8% of the patients [35]. Generally, the AEs were of lower grade and well-tolerated, indicating the safe nature of SABT.

As to limitations, the retrospective nature of this study and limited sample size, partly due to the rare morbidity, lowered the evidence level of this study. Even so, it provided preliminary evidence for SABT as a means to treat recurrent HNACC after surgery or EBRT. The evidence from prospective studies is warranted to further confirm the role of SABT in recurrent HNACC.

## 4. Materials and Methods

### 4.1. Patients

The patients diagnosed with recurrent HNACC after surgery or EBRT, undergoing I-125 seed SABT from May 2011 to November 2019, were retrospectively reviewed. Written informed consent was obtained from each patient and this study was approved by the Ethics Committee of our hospital.

### 4.2. Inclusion and Exclusion Criteria

The inclusion criteria are as follows: (1) confirmed HNACC by pathologic or imaging diagnosis; (2) disease > 1 cm and ≤ 7 cm; (3) medically inoperable or individual refusal to resection; (4) unfit for re-EBRT after a comprehensive evaluation by radiation oncologists or individual refusal to re-EBRT; (5) tolerate anesthesia and puncture; (6) suitable puncture access; (7) no severe tendency for bleeding; (8) Karnofsky performance status (KPS) ≥ 60; (9) adequate hematological reserves: white blood cell counts ≥ 3 × 109/mL; granulocyte counts ≥ 1.5 × 109/mL; hemoglobin ≥ 90 g/L; platelet > 50 × 109/L; (10) adequate hepatic function: total serum bilirubin concentration ≤ 1.5 times the upper limit normal (ULN); serum transaminases and alkaline phosphatase ≤ 2.5 ULN; (11) adequate renal function: serum creatinine concentration ≤1.5 ULN or creatinine clearance >50 mL/min; (12) adequate heart function: left ventricular ejection fraction (LVEF) ≥ 50%; (13) expected survival > 3 months.

The exclusion criteria are as follows: (1) unconfirmed head and neck mass; (2) any mental disorder; (3) anticoagulant or anti-aggregate therapies were discontinued for less than 1 week before SABT; (4) patients with active infectious disease, trauma, stroke and severe wounds; (5) other somatic comorbidities of clinical concern; (6) pregnancy and lactation, (7) patient consent withdrawal.

### 4.3. Treatment

**Figure 3:**
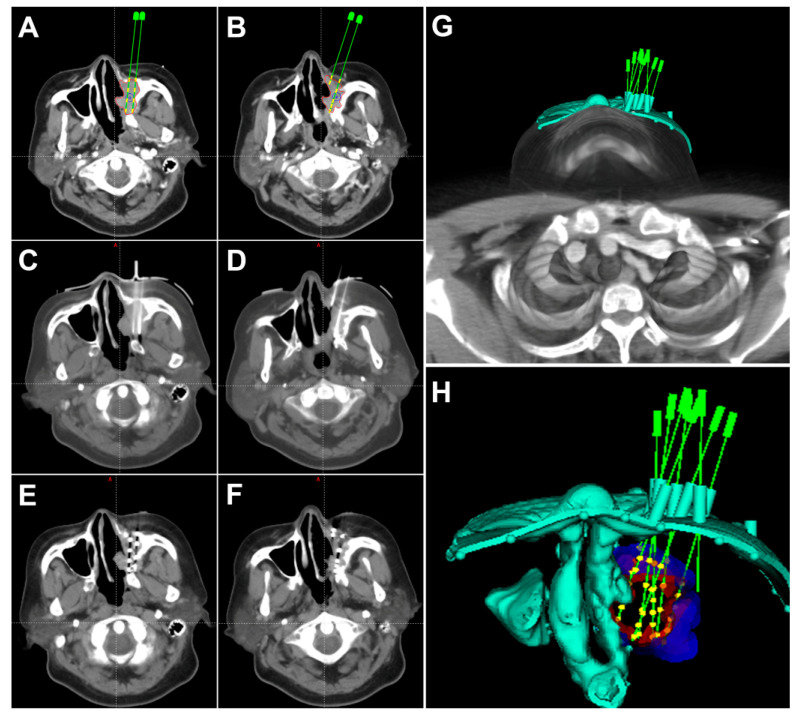
The CT images of pre-, intra- and post-operation. (**A**,**B**) show the preoperative treatment planning including the planned locations of needles, distribution of seeds and gross tumor volume (GTV). The green needles and yellow seeds are simulated needles and seeds in the brachytherapy treatment planning system (B-TPS), respectively. (**C**,**D**) show the actual locations of needles during operation. (**E**,**F**) show the actual postoperative distribution of seeds in target volume. (**G**,**H**) show the 3D-printing non-coplanar template (3D-PNCT) model with guide holes in the B-TPS.

The personalized SABT procedure mainly includes individualized preoperative treatment planning, intraoperative seed implantation and postoperative dosimetric evaluation (Figure 3).

### 4.4. Preoperative Individualized Treatment Planning

Patients undergo setup and CT simulation 2–3 days prior to the seed implantation to obtain three-dimensional information of tumor volume and organs at risk (OARs). Then we use the brachytherapy treatment planning system (B-TPS; Beijing Feitian Industries Inc. and Beijing University of Aeronautics and Astronautics, Beijing, China) to evaluate the feasibility of seeding. If feasible, the pretreatment plan is made by a medical physicist including the delineation of gross tumor volume (GTV) and adjacent OARs, determination of prescription dose, design of the accesses of the insertion needles (direction, distribution and depth) and spatial distribution of I-125 seeds, and calculation of the dose in the GTV and OARs. The dosimetric goal is that the dose received by 90% of the GTV (GTV D90) reaches the prescription dose as far as possible. The doses delivered to the OARs are as low as possible through optimization.

### 4.5. Intraoperative Implantation

Two different methods are used for intraoperative implantation. Method A is used for the patients before 2015 and method B is used for the patients after 2015 when the 3D printed non-coplanar template (3D-PNCT) was invented by our center (Patent No. ZL201620414011.9).

Method A: Implantation guided by CT without the assistance of the template. After re-setup, skin preparation, draping and local anesthesia, disposable needles (Mick Radio Nuclear Instruments, Mount Vernon, NY, USA) are inserted into the target volume under CT guidance along the puncture points on the skin which are designed preoperatively. I-125 seeds (CIAE-6711; Chinese Atomic Energy Science Institution, Beijing, China) are then implanted by the Mick applicator (Mick Radio-Nuclear Instruments Inc., Mount Vernon, NY, USA).

Method B: Implantation guided by CT with aid of the 3D-PNCT. After the treatment plan is designed in the B-TPS, a 3D personal template model is generated at once including the information of needle distribution and the characteristics of the therapeutic area outline (Figure 3G,H). The 3D-PNCT is then printed by the 3D light-cured rapid-forming printer. Then after re-setup, skin preparation, draping and local anesthesia, the 3D-PNCT is aligned to the surface of the therapeutic area according to the outline of the patient, positioning line on the patient, alignment line on the 3D-PNCT and laser. Then the implantation needles are inserted to the predetermined depth through the guide holes on the 3D-PNCT guided by CT. “Fine-tuning”, if necessary, is carried out. After insertion, the I-125 seeds are implanted into the tumor as mentioned above.

See our previous work [10] for the details of the seeding procedures.

### 4.6. Postoperative Evaluation

Following seed implantation, a CT scan is immediately conducted to validate the postoperative distribution of seeds. Then the images are loaded to B-TPS to calculate the actual seeds and dose distribution (Figure 3E,F). Dosimetric parameters including D90, D100, V100, V150, V200 are used to evaluate the dosimetry, where Dx indicates the dose delivered to x% of GTV, and Vx indicates the percentage of GTV receiving x% of the prescribed dose.

All physicians participating in the procedure of SABT are well trained and legally qualified. All procedures are performed in accordance with relevant guidelines and regulations.

### 4.7. Follow-Up

Patients are followed up every 3 months for the first 2 years, then every 6 months from 3 to 5 years and annually thereafter.

### 4.8. Clinical Outcomes

The clinical outcomes included the objective response rate (ORR), disease control rate (DCR), local control time (LCT), overall survival (OS), safety and symptom relief. Response Evaluation Criteria in Solid Tumors (RECIST) version 1.1 is used to evaluate the clinical responses including complete response (CR), partial response (PR), stable disease (SD), and progressive disease (PD). ORR is confirmed in cases with CR or PR and DCR is confirmed in cases with CR or PR or SD. LCT is defined as the time from SABT to local progression with OS defined as the time from SABT to death from any cause. The safety profiles include operation complications and radiation-related adverse events (AEs) assessed as per RTOG Common Toxicity Criteria.

### 4.9. Statistical Analysis

The parameters are expressed as categorical variables or continuous variables. The categorical variables are compared by the Chi-square or Fisher’s exact test, while continuous variables are compared by the t-test or rank-sum test. The LCT and OS are analyzed through the Kaplan–Meier method and compared using the log-rank test. Univariate analyses of LCT and OS are performed using the Cox proportional hazard regression model. *p* < 0.05 is considered as statistically significant. SPSS 24.0 software (SPSS, Chicago, IL, USA) is used for the statistical analyses.

## 5. Conclusions

For patients with recurrent HNACC after the failure of surgery and EBRT, SABT showed a good response rate, favorable local control and survival in accompany with a satisfying safety, which suggested that SABT can be an effective and safe alternative option for recurrent HNACC. The dose may influence the local control, and dose constraints could be defined in larger patient cohorts.

## Figures and Tables

**Table 1:** General information for the 21 patients in this study.

Parameter	Category	Cases (%)
Age	Median (range)	56 (11–75)
Sex	Male	8 (38.1)
	Female	13 (61.9)
KPS	60	1 (4.8)
	70	3 (14.3)
	80	7 (33.3)
	90	10 (47.6)
Tumor size (cm^3^)	<3 cm	9 (42.9)
	3–6 cm	11 (52.4)
	>6 cm	1 (4.8)
Prior treatment	None	0 (0.0)
	Surgery	17 (81.0)
	EBRT	16 (76.2)
	Surgery and EBRT	12 (57.1)
Prior surgery times	0	4 (19.0)
	1	15 (71.4)
	2	2 (9.5)
Prior radiotherapy times	0	5 (23.8)
	1	13 (61.9)
	2	3 (14.3)
Prior cumulative radiotherapy dose	<60 Gy	1 (4.8)
	≥60 Gy	15 (71.4)
GTV (cm^3^)	Median (range)	25.8 (4.2–63.5)
Prescription dose (Gy)	Median (range)	140 (90–180)
No. of needles	Median (range)	9 (2–19)
No. of Seeds	Median (range)	36 (8–80)
Seed activity (mCi)	Median (range)	0.59 (0.40–0.79)
D90 (Gy)	Median (range)	137.1 (81.0–178.5)

Abbreviations: EBRT, External Beam Radiotherapy; KPS, Karnofsky Performance Score; HNACC, Head and Neck Adenoid Cystic Carcinoma; GTV, Gross Tumor Volume.

**Table 2:** Univariate analyses for LCT.

Variables	Categories	Univariate Analyses		
		HR	95% CI	*p*-Value
Age(year)	>56	0.480	0.107–2.161	0.339
	≤56			
Sex	Female	1.248	0.270–5.761	0.777
	Male			
KPS	>80	0.228	0.043–1.205	0.082
	≤80			
Prior Surgery	Yes	0.774	0.147–4.071	0.762
	No			
Prior EBRT	Yes	1.587	0.185–13.625	0.674
	No			
GTV(cm^3^)	>25.8	1.037	0.229–4.688	0.963
	≤25.8			
D90(Gy)	>137.1	0.117	0.014–0.979	0.048
	≤137.1			

Abbreviations: LCT, Local control time; KPS, Karnofsky Performance Score; EBRT, External Beam Radiotherapy; HR, Hazard Ratio; CI, Confidence Intervals.

**Table 3:** Univariate analyses for OS.

Variables	Categories	Univariate Analyses		
		HR	95% CI	*p*-Value
Age (year)	>56	0.534	0.089–3.209	0.493
	≤56			
Sex	Female	3.290	0.361–30.016	0.291
	Male			
KPS	>80	2.796	0.308–25.367	0.361
	≤80			
Prior Surgery	Yes	0.537	0.073–3.940	0.541
	No			
Prior EBRT	Yes	0.860	0.087–8.458	0.897
	No			
GTV(cm^3^)	>25.8	6.022	0.664–54.734	0.111
	≤25.8			
D90(Gy)	>137.1	0.273	0.030–2.453	0.247
	≤137.1			

Abbreviations: OS, Overall Survival; KPS, Karnofsky Performance Score; EBRT, External Beam Radiotherapy; HR, Hazard Ratio; CI, Confidence Intervals.

## Data Availability

The data presented in this study are available on request from the corresponding author. The data are not publicly available due to privacy or ethical restrictions.
